# Correction: Díaz-García et al. *Candida* Genotyping of Blood Culture Isolates from Patients Admitted to 16 Hospitals in Madrid: Genotype Spreading during the COVID-19 Pandemic Driven by Fluconazole-Resistant *C. parapsilosis*. *J. Fungi* 2022, *8*, 1228

**DOI:** 10.3390/jof9020196

**Published:** 2023-02-03

**Authors:** Judith Díaz-García, Ana Gómez, Marina Machado, Luis Alcalá, Elena Reigadas, Carlos Sánchez-Carrillo, Ana Pérez-Ayala, Elia Gómez-García de la Pedrosa, Fernando González-Romo, María Soledad Cuétara, Coral García-Esteban, Inmaculada Quiles-Melero, Nelly Daniela Zurita, María Muñoz Algarra, María Teresa Durán-Valle, Aída Sánchez-García, Patricia Muñoz, Pilar Escribano, Jesús Guinea, on behalf of the CANDIMAD Study Group

**Affiliations:** 1 Clinical Microbiology and Infectious Diseases Department, Hospital General Universitario Gregorio Marañón, 28007 Madrid, Spain; 2 Instituto de Investigación Sanitaria Gregorio Marañón, 28007 Madrid, Spain; 3 CIBER Enfermedades Respiratorias-CIBERES (CB06/06/0058), 28029 Madrid, Spain; 4 Clinical Microbiology Department, Hospital Universitario 12 de Octubre, 28041 Madrid, Spain; 5 Instituto de Investigación Sanitaria del Hospital 12 de Octubre, 28041 Madrid, Spain; 6 Clinical Microbiology Department, Hospital Universitario Ramón y Cajal, 28034 Madrid, Spain; 7 Instituto Ramón y Cajal de Investigación Sanitaria (IRYCIS), 28034 Madrid, Spain; 8 CIBER de Enfermedades Infecciosas (CIBERINFEC), Instituto de Salud Carlos III, 28029 Madrid, Spain; 9 Clinical Microbiology Department, Hospital Universitario Clínico San Carlos, 28040 Madrid, Spain; 10 Instituto de Investigación Sanitaria del Hospital Clínico San Carlos IdISSC, 28040 Madrid, Spain; 11 Clinical Microbiology Department, Hospital Universitario Severo Ochoa, 28914 Leganés, Spain; 12 Clinical Microbiology Department, Hospital Universitario de Getafe, 28901 Madrid, Spain; 13 Clinical Microbiology Department, Hospital Universitario La Paz, 28046 Madrid, Spain; 14 Clinical Microbiology Department, Hospital Universitario de La Princesa, 28006 Madrid, Spain; 15 Clinical Microbiology Department, Hospital Universitario Puerta de Hierro Majadahonda, 28220 Madrid, Spain; 16 Clinical Microbiology Department, Hospital Universitario de Móstoles, Móstoles, 28935 Madrid, Spain; 17 Laboratorio Central de la CAM-URSalud-Hospital Infanta Sofía, San Sebastián de los Reyes, 28703 Madrid, Spain; 18 Medicine Department, Faculty of Medicine, Universidad Complutense de Madrid, 28040 Madrid, Spain

## Error in Figure 2:

In the original publication [1], a format error in Figure 2 was detected. A correction has been made to Figure 2 in the Results section, Section 3.1. The legend to the figure was truncated, and the missing parts made the interpretation of the whole figure difficult. Figure 2 should be changed to:

The authors apologize for any inconvenience caused and state that the scientific conclusions are unaffected. This correction was approved by the Academic Editor. The original publication has also been updated.

## Figures and Tables

**Figure 2:**
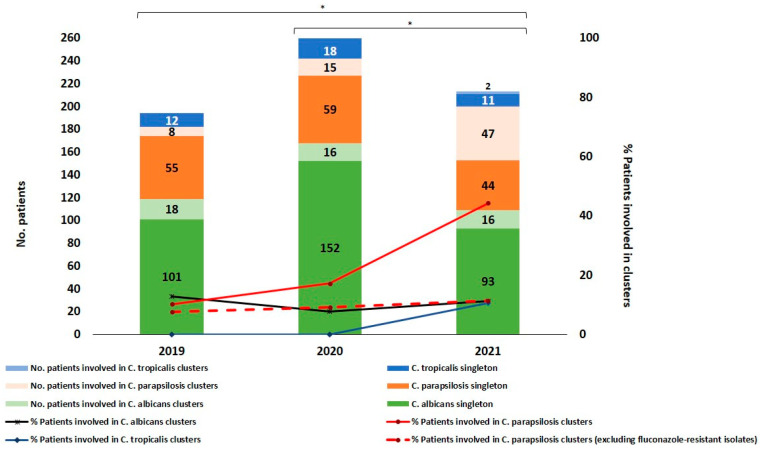
Numbers (and percentage) of patients involved in singleton and intra-hospital clusters per species over the study period. * Differences reaching statistical significance (*p* < 0.05).
